# Large Infants

**Published:** 1915-10

**Authors:** 


**Large Infants.** A. R. Bisot, Louisville, Paediatrics, Aug. The average weight at birth is 7.75 lbs. The following instances of small infants at term are recorded: Sir Edward Home, 1824, 1 lb., lived to age of 9 years; Moore 1880, 1½ lbs., well at 15 months when the weight was 18 lbs.; Mursick, 1874, 1¾ lbs., living and well; Michigan twins 1874, 1½ and 1¾ lbs., length 8 inches, active and well; Samaritan Hospital, Philadelphia, 1896, 1¾ lbs. In 3,600 births at the Rotunda Hospital, Dublin, only one infant reached the weight of 11 lbs Among older writers, Cranz reports an infant weighing 23 lbs., Fern 18, Mittehauser 24, von Siebold 22½. Warren, 1884, reports twins weighing respectively 17½ and 18 lbs., the placenta weighing 4 lbs., and there being "an ordinary pailful" of liquor amnii. Meadows, 1860, and Burgess, 1875, report weights of 18 lbs. Eddowes, 1884, 20⅛ lbs.; Chubb, 1879, 21; Dickinson, 1894, 9, 20 and 16 lbs. as the weights of the successive infants of the same mother; Rice, 1878, 20¼; Johnston, 1881, and Smith, 1878, 20; Baldwin, 1894, 23 lbs., following three miscarriages. Beach, 1879, states that the giant Bates had a child delivered after a slow labor, weighing 23¾ lbs. and 30 inches long. The secundines weighed 10 lbs., and the amniotic fluid measured 9 quarts. Various other cases are cited.

